# Anti-inflammatory treatment strategies for ischemia/reperfusion injury in transplantation

**DOI:** 10.1186/1476-9255-7-27

**Published:** 2010-05-28

**Authors:** Jens Lutz, Klaus Thürmel, Uwe Heemann

**Affiliations:** 1 Department of Nephrology, II. Medizinische Klinik, Klinikum rechts der Isar, Technische Universität München, Germany

## Abstract

Inflammatory reactions in the graft have a pivotal influence on acute as well as long-term graft function. The main reasons for an inflammatory reaction of the graft tissue are rejection episodes, infections as well as ischemia/reperfusion (I/R) injury. The latter is of particular interest as it affects every solid organ during the process of transplantation. I/R injury impairs acute as well as long-term graft function and is associated with an increased number of acute rejection episodes that again affect long-term graft outcome.

I/R injury is the result of ATP depletion during prolonged hypoxia. Further tissue damage results from the reperfusion of the tissue after the ischemic insult. Adaptive cellular responses activate the innate immune system with its Toll-like receptors and the complement system as well as the adaptive immune system. This results in a profound inflammatory tissue reaction with immune cells infiltrating the tissue. The damage is mediated by various cytokines, chemokines, adhesion molecules, and compounds of the extracellular matrix. The expression of these factors is regulated by specific transcription factors with NF-κB being one of the key modulators of inflammation.

Strategies to prevent or treat I/R injury include blockade of cytokines/chemokines, adhesion molecules, NF-κB, specific MAP kinases, metalloproteinases, induction of protective genes, and modulation of the innate immune system. Furthermore, preconditioning of the donor is an area of intense research. Here pharmacological treatment as well as new additives to conventional cold storage solutions have been analyzed together with new techniques for the perfusion of grafts, or methods of normothermic storage that would avoid the problem of cold damage and graft ischemia.

However, the number of clinical trials in the field of I/R injury is limited as compared to the large body of experimental knowledge that accumulated during recent years in the field of I/R injury. Future activities in the treatment of I/R injury should focus on the translation of experimental protocols into clinical trials in order to reduce I/R injury and, thus, improve short- as well as long-term graft outcome.

## Introduction

Inflammatory reactions fundamentally influence the short-term as well as the long-term performance of solid organ allografts. Thus, it is crucial to control such inflammatory reactions in order to improve graft function as well as allograft survival. Inflammatory reactions are differentially initiated in a graft following transplantation. Important reasons for an inflammatory reaction of the graft are alloantigen directed immune reactions of the recipient resulting in rejection episodes with heavy inflammation of graft tissue. On the other hand the transplant procedure with its related ischemia/reperfusion (I/R) injury and the surgical trauma itself could result in acute as well as chronic inflammatory reactions that influence allograft function over the long-term [1]. This review will focus particularly on the mechanisms related to inflammatory reactions following ischemia/reperfusion injury in the transplant setting and strategies for the prevention as well as the treatment of I/R injury.

## Molecular Mechanisms involved in the Development of Tissue Injury after Ischemia/Reperfusion

Different mechanisms participating in the development of ischemia reperfusion injury will be reviewed in the following section. I/R injury is the result of a prolonged oxygen deprivation in a tissue leading to hypoxia. This results in an ATP-depletion of the cells leading to swelling of mitochondria eventually causing a release of cytochrome c from the mitochondria. Cytochrome c activates an apoptotic signaling cascade involving caspases 1 and 9. These events participate in the induction of an inflammatory response via generation of IL-1β as well as programmed cell death (apoptosis) by activation of different caspases. Moreover, ATP depletion induces a cellular edema that occurs particularly during cold ischemia when Na/K ATPase is inhibited [2].

A crucial mediator of I/R injury are oxygen derived free radicals [3]. Particularly hydrogen peroxide, a source of oxygen-derived free radicals after hypoxia, can induce TNF-α by an activation of p38 mitogen activated kinase (MAPK) [4]. Additionally, a number of intracellular adaptive metabolic responses occur among them an increase in the intracellular Ca^++^-concentration with generation of calcium pyrophosphate complexes and the formation of uric acid. Calcium phosphate complexes and uric acid that belong to a group of so called danger signals (DNA fragments, cell membrane fragments, heat shock proteins, etc.) can bind to intracellular protein complexes so called inflammasomes [5]. The inflammasomes include different adaptor molecules that mediate an increase of the production and secretion of interleukin-1 (IL-1)β. Furthermore also Toll-like receptors are stimulated through danger signals eventually stimulating the secretion of further proinflammatory cytokines/chemokines through an activation of NF-κB [6].

The transcription factor NF-κB plays a central role in the generation of an inflammatory response as it is activated under conditions of cell stress and inflammation resulting in an activation and formation of other pro-inflammatory factors such as IL-1β, tumor necrosis factor (TNF)-α, or interferon (IFN)-γ and chemokines such as IL-8, MCP-1, or RANTES potentiating the inflammatory response. This is followed by an infiltration of lymphocytes, mononuclear cells/macrophages, and granulocytes into the injured tissue. Here adhesion molecules like the leukocyte function associated antigen-1 (LFA-1) or the intercellular adhesion molecule (ICAM)-1 play an important role. The cellular infiltrate together with the expression of cytokines/chemokines aggravates the interstitial edema of the inflamed tissue.

Apart from the formation of calcium phosphate complexes, the increase of the intracellular calcium concentration also enhances the activation of phospholipases as well as proteases. The latter group includes calpains (cleaving protein kinase c, fodrin, components of the cytoskeleton) and caspases which execute programmed cell death (apoptosis).

An important effect of hypoxia on a tissue is the development of metabolic acidosis. It occurs as a result of hypoxia when anaerobic glycolysis is the only way to generate energy. However, it can induce an inflammatory response when perfusion of the respective tissue is restored after hypoxia as well as hypothermia [7].

However, not only locally generated inflammatory mediadtors like cytokines/chemokines [8] resulting from I/R injury but also systemic inflammatory mediators in the donor affect the graft after transplantation. Here brain death profoundly contributes to a systemic inflammatory response through the release of cytokines from the brain. This "cytokine storm" deteriorates organ function resulting in more acute rejection episodes and decreased long-term function [9-11]. The significance of such effects is underlined by experiments demonstrating the deleterious influence of brain death on graft function also over the long-term [12] even when cold ischemia has been eliminated [13].

If the inflammation is resolved the tissue can heal without sequale. However, if the inflammatory response is not resolved, for example due to ongoing tissue injury, the inflammation can become chronic, thus, stimulating tissue remodeling that eventually can result in organ fibrosis with a consecutive loss of function and graft failure [1]. Fibrosis with an accumulation of extracellular matrix is a late non-specific result after I/R injury. However, extracellular matrix breakdown plays also a role in mediating acute tissue injury after I/R as discussed below. Furthermore, components of the innate immune system such as Toll-like receptors or the complement system also participate in the development of I/R injury.

Evidence exists that the temperature during ischemia differentially affects tissue injury as in liver ischemia for example cold ischemia (time when the graft is outside the body during transport from the donor to the recipient) affects more the sinusoidal cells while warm ischemia (time when the graft is in the body during operation before perfusion is reinstalled) affects primarily the hepatocytes [14,15].

On the other hand cells do not only produce deleterious factors promoting cell death and inflammation during hypoxia but also form protective factors in order to survive hypoxic episodes. Here the transcription factor HIF (hypoxia inducible factor)-1 plays an important role [16]. Interestingly, the HIF-1 system may not only be activated under hypoxic conditions but also under inflammatory conditions [17]. Basically cellular HIF-1 levels are low under normoxic conditions while they increase under hypoxic conditions to increase angiogenesis, erythropoesis, vasomotor control of the vessels and alterate the cellular energy metabolism as well as survival programs in order to protect the cells from the effects of hypoxia. Apart from transcription factors also protective genes like hemogygenase-1, bcl-2 or A20 are induced to protect cells after hypoxia [18].

Reducing or preventing ischemia/reperfusion injury is a central strategy for an improvement of short-term as well as long-term graft performance after transplantation.

Evidence for this concept is derived from the observations that organs from living donors have a better graft survival and graft performance than organs from cadaveric donors although the HLA mismatch is similar because the ischemia times are significantly shorter in the setting of living donor related transplantation [19,20].

Strategies to reduce I/R injury after solid organ transplantation can be divided into pretransplant- and posttransplant strategies. Pretransplant strategies for the reduction of I/R injury include reduction of cold ischemia time through a logistic optimization of graft transport, machine-based perfusion procedures, or optimization of preservation solutions.

A further strategy is preconditioning of the donor with substances that have the capacity to reduce I/R injury. Posttransplant strategies to reduce I/R injury include the administration of substances that interfere with the inflammatory process mainly with the action of chemokines, cytokines, or leukocyte infiltration. On the other hand, strategies interfering with programmed cell death (apoptosis) have been investigated as well in order to reduce cell death and thus, protect the graft from cell loss. An ideal intervention would be a short course of a treatment during the immediate peritransplant period followed by a long-lasting effect in order to spare medications with their potential side effects. One practical problem in the prevention/treatment of I/R injury is that many treatment options were successful in experimental models while only few have been introduced into clinical trials not to mention standard treatment protocols. Here much work has to be done in the future to convert the knowledge derived from experimental approaches into successful clinical practice. In this review we will focus on the different experimental strategies to interfere with I/R damage and discuss strategies that have been already introduced into clinical practice.

## Therapeutic targets and strategies to interfere with I/R injury

### Chemokines/Cytokines

Most pro-inflammatory chemokines such as IL-8, MCP-1, ENA-78, MIP-1α, MIP-1β, or RANTES require activation by transcription factors such as nuclear factor (NF)-κB or activating protein (AP)-1 [8]. A central kinase required for the activation of these transcription factors is p38 MAPK. Indeed, inhibition of this kinase reduced pro-inflammatory cytokine production and I/R injury in an experimental model of hypoxia [21].

The generation of chemokines is increased already a few hours after transplantation. Inhibition of caspases, particularly caspase-1, NF-κB, p38 MAPK all effectively ameliorated I/R injury by reducing the generation of chemokines in experimental models [8]. However, in order to reduce chemokine effects it is not enough to only reduce their production. Their effects have also to be blocked. A promising molecule for such purposes is Met RANTES, a derivative of the chemokine RANTES, that blocks the actions of RANTES at the receptor level. Treatment with Met-RANTES at an early time period from day 0 to day 10 after transplantation, when ischemia/reperfusion injury is present, reduced the development of fibrosis in the graft in an experimental rat transplant model [22]. This beneficial effect is supposed to be due to a reduced inflammatory reaction after transplantation resulting in better graft outcome over the long-term. Similar effects have been achieved with blockade of MCP-1 and MIP in experimental models of I/R injury [8,23].

Apart from chemokines also cytokines play a crucial role in mediating I/R injury after transplantation. An important group of pro-inflammatory cytokines is the interleukin-1 family. Here particularly IL-1β is of interest as it can link the effects of the innate immune system, particularly the activation of Toll-like receptor 4, with the cellular adaptive immune response [24]. IL-1β induces the expression of adhesion molecules on endothelial cells, thus, facilitating cellular infiltration. Furthermore it induces the production of prostaglandins through an increased expression of cyclooxygenases and increases the number of circulating neutrophils and thrombocytes. The action of IL-1 is physiologically inhibited by IL1-RA (receptor antagonist) [25]. We analyzed the effects of IL-1 RA on the development of renal I/R injury in an experimental rat model [26]. Animals treated with IL-1RA had a significantly lower I/R damage as well as reduced inflammatory infiltrates and number of apoptotic cells as compared to untreated controls. In these experiments a substantial reduction of I/R damage was achieved with compounds that have been introduced into clinical practice as IL-1 RA is available for the treatment of rheumatoid arthritis.

### Adhesion molecules

Adhesion molecules, in particular the leukocyte function associated antigen-1 (LFA-1) and the intercellular adhesion molecule (ICAM)-1, are necessary for the infiltration of immune cells from the vessel lumen into the tissue. Experimental evidence suggests that a reduced expression of adhesion molecules ameliorates the development of I/R injury [27-30] after transplantation.

LFA-1 has various functions in immune reactions among them adhesion and trafficking of leukocytes, stabilization of the MHC-T cell receptor complex as well as providing costimulation signals. In a clinical study efalizumab, a humanized IgG1 anti-LFA-1 antibody, was administered to recipients of kidney grafts after transplantation with a good tolerability [31]. However, information on long-term effects on the grafts as well as the influence of this treatment on I/R injury are missing so far as the study was aimed to analyze calcineurin inhibitor sparing treatment protocols.

Another important adhesion molecule is ICAM-1. In a phase I/II study an anti-ICAM-1 antisense-oligonucleotide was analyzed in order to prevent acute rejection episodes. Altogether the oligonucleotide did not further reduce the rate of acute rejections or improved graft survival as compared to a conventional immunosuppressive protocol [32]. Anti-adhesion molecule directed therapies could be of benefit in the transplant setting; however, more data is needed before the clinical significance of this therapeutic approach can be evaluated.

### Interventions inhibiting NF-κB

The IKK complex is a key regulator of IκB degradation and, thus, NF-κB activation [33]. Specific IKK complex antagonists reduced I/R injury in the setting of experimental myocardial infarction [34]. However, this approach warrants further investigation in the setting of transplantation.

Another way to inhibit IκB degradation would be the inhibition of proteasomes that are responsible for a breakdown and thus, termination of function, of specifically marked proteins. In renal, cerebral as well as hepatic ischemia the respective injury could be prevented if the proteasome inhibitor lactacystin or its derivative PS519 were administered before the initiation of ischemia [35-37].

Experimental protocols have been introduced that analyzed the effects of gene therapy, inhibition of NF-κB nuclear translocation as well as oligodeoxynucleotide interference with NF-κB [33]. However, all these approaches have not been translated into larger clinical trials so far.

### Innate immune system

#### Toll-like receptors

It has been demonstrated that a genetic deletion of the Toll-like receptor 2 [38] and the Toll-like receptor-4 [39] in experimental ischemia/reperfusion models resulted in a significantly reduced tissue injury as compared to controls. Using adoptive transfer it has also been demonstrated that the missing expression of Toll-like receptors on the injured tissue rather than on the infiltrating immune cells is the responsible mechanism for the protective effects. This fits to recent clinical observations where grafts with defective TLR-4 signaling had a better function and lower expression of pro-inflammatory cytokines after transplantation than grafts with regular TLR-4 signaling [40]. In our experimental I/R model a double deletion of the TLR- 2 together with the TLR-4 did not result in an additional protective effect as compared to the single deletions [41]. Thus, MyD88 independent signal pathways do not seem to play an important role for the development of an I/R injury in this model. Experimental evidence exists that also TLR antagonists like glucan phosphate or the synthetic LPS analogue eritoran can prevent I/R injury in models of experimental myocardial ischemia [42,43].

#### Complement system

The complement system consists of various components that are activated upon different stimuli such as infectious agents like bacteria, viruses, or non-infectious stimuli among them I/R injury [44-46]. Basically, activation of the complement system leads to a binding of complement factors to certain targets such as bacteria or injured cells, chemotaxis as some components can attract inflammatory cells to the site of injury, and destruction of target cells by distinct lytic mechanisms. The inhibition of the complement system has been proven effective in reducing I/R injury in experimental models [47,48]. Targeting of the complement component C5 reduced I/R injury after transplantation in an experimental model [49]. However, targeting of the complement system to reduce I/R injury in clinical settings needs to be analyzed in the future.

### Protective genes

Cells possess a number of so called protective genes. The expression of such genes is enhanced during periods in which the cell is at danger due to otherwise detrimental stimuli/influences and protects them by an inhibition of programmed cell death (apoptosis) and inflammatory responses (i.e. inhibition of NF-κB). Overexpression of Bcl-2 [50-53] or hemoxygenase-1 (HO-1) [54-57] through replication-defective viral vectors significantly reduced tissue damage in different experimental models of I/R injury as well as transplantation.

The inhibition of apoptosis seems to be of importance in tissue protection as caspase inhibitors also substantially reduced I/R injury together with a marked reduction of the inflammatory response [58]. Thus, inhibition of apoptosis not only prevents loss of cells but also seems to be associated with a reduction of inflammation. On the other side substances known to promote apoptosis like mTOR inhibitors have been demonstrated to aggravate I/R injury when given in the early phase after an ischemic insult to the kidney [59,60].

#### Zink finger protein A20

A molecule with potential protective effects is the zink finger protein A20 as it combines anti-apoptotic as well as anti-inflammatory effects [61]. It has ubiquitinating and de-ubiquitinating properties, thus, activating and/or inhibiting target molecules [62,63] which modulate regulatory adapter molecules. It regulates receptor mediated apoptosis (for example through TNF-α) by modulation of caspase-8 and exerts anti-inflammatory effects through an inhibition of Nf-κB as well as Toll-like receptor 4 signaling [64]. We analyzed the over-expression of A20 in a hepatic as well as a renal I/R model [65]. Overexpression of A20 resulted in a significantly reduced I/R injury in both models that was mediated by a decreased activation of NF-κB as well as a reduced expression of adhesion molecules. In a liver transplant model the overexpression of A20 resulted in a better graft regeneration as well as in reduced graft rejection with improved graft survival [66]. So far, no clinical experiences regarding the protective effect of A20 overexpression in the transplant setting exist.

#### Serum and glucocorticoid regulated Kinase 1

The serum- and glucocorticoid-inducible protein kinase 1 (SGK1) is a serine-threonine protein kinase activated through the phosphatidylinositol 3-kinase (PI3-kinase) pathway counteracting apoptosis [67]. Expression and activation of SGK1 are increased in various models of cell stress [68-70]. Hypoxia/reoxygenation increased SGK1 transcript levels, SGK1 protein abundance and SGK1 phosphorylation *in vitro*. Furthermore, hypoxia/reoxygenation enhanced the percentage of apoptotic cells, an effect significantly blunted by prior overexpression of SGK1.

*In vivo *experiments of renal I/R injury demonstrated increased SGK1 transcript levels and SGK1 protein abundance in renal tissue. The ischemia was followed by enhanced apoptosis, an effect significantly more pronounced in gene targeted mice lacking SGK1. Thus, SGK1 is up-regulated following hypoxia/reoxygenation *in vitro *and ischemia *in vivo *and counteracts apoptosis. It has to be analyzed in the future whether such effects can be demonstrated also in the transplant setting.

### The extracellular matrix during I/R injury

Extracellular matrix turnover influenced by matrix metalloproteinases seems to play an important role for tissue remodeling after ischemia/reperfusion injury. Particularly the matrix metalloproteinases 2, 3, and 9 have been shown to influence tissue repair. In experimental models of I/R injury an inhibition of MMPs significantly reduced tissue damage [71-73].

Experiments of our group in a chronic rat kidney transplantation model demonstrated that, as expected, animals receiving delayed treatment (week 12-20 after transplantation) with the matrix metalloproteinase inhibitor BAY 12-9566 developed severe fibrosis and proteinuria as compared to non-treated animals [22]. However, when BAY 12-9566 was administered early (day 0-10 after transplantation), tissue damage was reduced with better graft performance. This provides evidence for the concept that an early time restricted intervention of an inflammatory process would have long lasting positive effects although the specific treatment has already been terminated. The reason for the beneficial effect of an early MMP-inhibition after transplantation could be a reduced degradation of basal membranes as well as other extracellular matrix components resulting in a preserved tissue structure as well as a reduced generation of chemotactic substances reducing tissue infiltration with inflammatory cells.

### Strategies of organ preservation

#### Cold preservation

Cold preservation is to date the standard procedure in reducing graft damage after ischemia and reperfusion. Strategies to add new additives to existing storage solutions or the development of completely new storage solutions, respectively, are under investigation [74]. Additives to cold storage solutions such as the p38 MAPK inhibitor FR 167653, the colloid polyethylene glycol, that reduces ATP depletion and inhibits calcium accumulation within the cells, have been successfully used in order to reduce I/R damage after transplantation [75-77]. However, the problem of cold injury itself, the difficulty to assess organ viability during cold storage and the development of I/R injury all make passive cold storage difficult for future improvements of strategies.

#### Donor preconditioning

Basically, the above mentioned strategies to modulate chemokines/cytokines, NF-κB, and protective genes can not only be applied in the recipient but also in the donor. However, in clinical settings this poses some ethical/legal as well as medical problems. It must be clarified that the interventions to protect against the development of I/R injury are not only helpful for the target organ but are also not harmful for the other potentially transplantable organs. Furthermore, donor preconditioning might be more effective if it would be initiated even before the diagnosis of brain death is established. However, this could be rather difficult from an ethical point of view.

On this note, a study of donor preconditioning with dopamine has been recently published [78]. In this randomized controlled trial the authors treated donors after the diagnosis of brain death with dopamine which resulted in a better acute graft outcome with reduced need for dialysis post-transplant although graft survival did not differ significantly between the groups. The authors suggested that these effects could be related to the protection of endothelial cells during cold storage from oxidative stress as hypothermic cell death and intracellular calcium accumulation are reduced by dopamine.

#### Hypothermic machine perfusion

Hypothermic ex vivo perfusion of the graft using a perfusion machine is under investigation since many years. Clinical studies showed that a better graft function with a reduced rate of delayed graft function after machine perfusion [79,80] which is in line with a metaanalysis suggesting a better graft outcome and cost effectiveness over the long-term after hypothermic machine perfusion as compared to the standard procedure of cold graft storage [81]. However, future research must demonstrate the practical clinical value of this approach.

#### Normothermic preservation

Normothermic preservation would have the potential to overcome the mentioned problems of cold storage, I/R injury, cold injury and problems to assess graft viability during storage [74]. Normothermic preservation has not only the potential to preserve organs but also to provide a basis for better recovery of graft function as compared to hypothermic preservation methods. The basis of the technique is to provide a physiological environment for the graft during the storage period using special perfusion solutions. It can be performed in multiple ways including blood based techniques with oxygenation. It has been used in the setting of heart, kidney, and liver transplantation with most experiences available in liver transplantation while it still has to find its way into clinical practice [82-84].

## Summary

Ischemia/reperfusion injury belongs to the main reasons for inflammatory reactions in solid organ allografts with profound influence on acute as well as long-term graft function. During recent years the interplay of the innate immune system with the adaptive immune system has become clearer for the pathogenesis of I/R injury. This is mediated by a number of cytokines/chemokines, adhesion molecules, transcription factors, and other pro-inflammatory compounds which can be specifically blocked resulting in a profound reduction of I/R injury. However, most of these approaches have not been transferred into clinical trials or even clinical treatment protocols. To date preservation techniques in the field of cold storage are available for clinical use while techniques for normothermic storage are intensely studied. Future research will concentrate on donor pretreatment/preconditioning as this would make prevention of I/R injury feasible.

## Competing interests

The authors declare that they have no competing interests.

## Authors' contributions

JL, KT, and UH contributed to the writing of this review. All authors read and approved the final manuscript.
